# Boosting Piezo/Photo-Induced Charge Transfer of CNT/Bi_4_O_5_I_2_ Catalyst for Efficient Ultrasound-Assisted Degradation of Rhodamine B

**DOI:** 10.3390/ma14164449

**Published:** 2021-08-09

**Authors:** Yang Wang, Dongfang Yu, Yue Liu, Xin Liu, Yue Shi

**Affiliations:** 1 Chang Wang School of Honors, Nanjing University of Information Science and Technology, Nanjing 210044, China; wangyang-cw@nuist.edu.cn (Y.W.); shiyue@nuist.edu.cn (Y.S.); 2 School of Environmental Science and Engineering, Nanjing University of Information Science and Technology, Nanjing 210044, China; liuy@nuist.edu.cn (Y.L.); lx@nuist.edu.cn (X.L.)

**Keywords:** piezocatalysis, CNT/Bi_4_O_5_I_2_, mechanical vibration, charge transfer

## Abstract

Strain-induced internal electric fields present a significant path to boosting the separation of photoinduced electrons and holes. In addition, piezo-induced positive/negative pairs could be released smoothly, taking advantage of the excellent electroconductibility of some conductors. Herein, the hybrid piezo-photocatalysis is constructed by combining debut piezoelectric nanosheets (Bi_4_O_5_I_2_) and typical conductor multiwalled carbon nanotubes (CNT). The photocatalytic degradation efficiency that the hybrid CNT/Bi_4_O_5_I_2_ exhibits was remarkably increased by more than 2.3 times under ultrasonic vibration, due to the piezo-generated internal electric field. In addition, the transient photocurrent spectroscopy and electrochemical impedance measurement reveal that the CNT coating on Bi_4_O_5_I_2_ enhances the piezo-induced positive/negative migration. Therefore, the piezocatalytic activity of CNT/Bi_4_O_5_I_2_ could be improved by three times, compared with pure Bi_4_O_5_I_2_ nanosheets. Our results may offer promising approaches to sketching efficient piezo-photocatalysis for the full utilization of solar energy or mechanical vibration.

## 1. Introduction

In recent years, piezoelectricity has been paid increasing attention, owing to its great potential in addressing environmental pollution and the energy crisis [1,2,3,4,5]. Piezoelectric crystals have the capacity to install an electric charge in reply to applied mechanical strain [6]. From the viewpoint of utilizing this mechanical stress, several kinds of promising piezocatalysts have been explored, including BaTiO_3_ [7,8], KNbO_3_ [9], PbTiO_3_ [10], ZnO [11], BiOBr [12], MoS_2_ [13], WSe_2_ [14], Bi_4_NbO_8_X (X = Cl, Br) [15], Bi_2_WO_6_ [16]. In particular, Wu et al. reported that the few-layers MoS_2_ displays high piezoelectric potential and ultrahigh catalytic performance [17]. Moreover, the piezo-induced electric field causes the edge of the conduction band of BiFeO_3_ to be higher than the H^+^/H_2_ potential, to efficiently generate H_2_ under ultrasonic vibration [18]. Nevertheless, the conversion efficiency of mechanical strain to an electric charge has often been limited by the low piezoelectric coefficient, poor electroconductibility, and unsatisfactory morphology [19,20,21,22]. Therefore, exploring efficient piezocatalysts and how to create modification strategies (e.g., heterostructure design, ion doping, noble metal deposition, defect engineering) have become significant solutions [8,23,24]. On the other hand, because of the resistance on the interaction between the liquid and solid phase, the release of piezo-induced positive/negative charges is limited to a certain extent, and still lacks the basic realization in practice of the positive/negative pairs [25].

Currently, photocatalytic technology is also a very promising route to controlling environmental pollution and satisfying the growing requirements for fossil fuel [26]. Still, photocatalytic efficiency has often been restricted by a poor solar response, ineffective carrier diffusion, and low stability [27,28,29]. Now, the catalytic activities of photocatalytic semiconductors can be efficiently tuned by piezo-induced internal electric fields, namely, the piezo-photocatalyst [30,31]. The piezo-photocatalyst is the multifield coupling between piezoelectricity and photoexcitation in semiconductors [9]. Primarily, the transfer of the photoinduced e^-^/h^+^ pairs could be boosted by the strain-induced internal electric fields. Despite that, it is still necessary to enhance the coupling efficiency of piezo-/photo-electricity.

In this work, novel piezo-photocatalyst Bi_4_O_5_I_2_ nanosheets are created. Bi_4_O_5_I_2_ nanosheets exhibit effective piezo-degradation ability, which was further improved with the addition of CNT for degrading Rhodamine B (RhB), due to the piezo-generated positive/negative pairs under ultrasonic vibration. In addition, hybrid CNT/Bi_4_O_5_I_2_, as a new piezo-photocatalyst, shows dramatically efficient degradation activity under the ultrasonic wave and simulated solar light, owing to the strain-induced internal electric field via the piezoelectric effect, which can boost the separation of photoinduced electron/hole pairs.

## 2. Experimental

### 2.1. Preparation of Catalysts

Pure Bi_4_O_5_I_2_ nanosheets were prepared by a solvothermal method, based on the previous report [32]. Typically, 5 mmol Bi(NO_3_)_3_∙5H_2_O and 10 mmol KI powders were dissolved into 80-mL ethylene glycol under continuous stirring for 30 min. Subsequently, the pH of the above suspension was adjusted to 10 by adding NaOH solution. Then the mixture was placed in a 100-mL Teflon-lined autoclave and kept at 150 °C for 12 h. After cooling to ambient temperature, the prepared products were separated using centrifugation, followed by washing with deionized water and ethanol three times, finally being kept at 60 °C for 10 h.

CNT/Bi_4_O_5_I_2_ was prepared by a solvothermal method similar to that of Bi_4_O_5_I_2_. Typically, a certain amount of pristine CNT (5%, 10%, 15%, 20%), 5 mmol Bi(NO_3_)_3_∙5H_2_O, and 10 mmol KI powders were dissolved into 80 mL of ethylene glycol under continuous stirring for 30 min. Subsequently, the pH of the above suspension was adjusted to 10 by adding NaOH solution. Then the mixture was placed in a 100-mL Teflon-lined autoclave and kept at 150 °C for 12 h. After cooling to ambient temperature, the prepared products were separated using centrifugation, followed by washing with deionized water and ethanol three times, finally being kept at 60 °C for 10 h.

### 2.2. Characterization

A powder X-ray diffractometer (MiniFlex 600, Rigaku, Japan) was used to ensure the crystal structure of synthesized samples, with Cu K_α_ radiation (λ = 0.15418 nm). The XRD patterns were determined at 5°/min from 10° to 80° (2θ). The morphology and structures of the catalysts were characterized by transmission electron microscope (TEM) and high-resolution transmission electron microscopy (HRTEM), using an FEI Talos F200X electron microscope (Thermo Fisher Scientific, Waltham, MA, USA) with an acceleration voltage of 200 kV. X-ray photoelectron spectroscopy (XPS) measurements were used to analyze the chemical compositions of different elements. All the binding energies were adjusted to the C1s peak at 284.8 eV. The UV-vis diffused reflectance spectrum (DRS) of the samples was determined with a UV-3600 plus spectrophotometer (Shimadzu, Kyoto, Japan) from 200 to 800 nm, with the BaSO_4_ as a reflectance standard.

### 2.3. Evaluation of Piezo-/Piezophoto-Catalytic Activities

The piezo-/piezophoto-catalytic performances of Bi_4_O_5_I_2_ based catalysts were probed by Rhodamine B (RhB). An optical fiber (300 W Xe lamp, BBZM-I) was used as the solar light source. An ultrasonic bath (80 W, AK-009A) with a frequency of 40 kHz was used to apply periodic local mechanical strain to the catalysts. At this point, 50 mg of samples were put into a 50-mL RhB aqueous solution (5 ppm). The mixture was stirred for 1 h to reach the equilibrium of adsorption-desorption in the dark. Then, the suspension was degraded by simulated solar light or mechanical strain. Afterward, 4 mL of the RhB solution was taken and centrifuged at intervals during the degradation process. Subsequently, the concentration of the supernatant was determined with a UV-visible spectrophotometer (721, Shanghai Jinghua, Shanghai, China).

In the trapping test, isopropyl alcohol (IPA), EDTA-2Na, and benzoquinone (BQ) dissolved by distilled water were used as scavengers to trap •OH, holes, and •O_2_^−^, respectively. When the catalyst was put into the pollutant solution, a certain amount of capture agent is added for the subsequent photocatalytic degradation process. The concentration of the capture agent IPA and EDTA-2Na is 1 mM, and that of the capture agent BQ is 0.1 mM. Finally, by comparing the effects of different capture agents on the degradation efficiency of pollutants, the main active substances that may exist in the degradation process were speculated.

The kinetics rates (*k*) were calculated by the following equation:

(1) $$ln\left(\frac{C_{0}}{C_{t}}\right)=kt$$

*C_t_* and *C_0_* are the concentrations of pollutants when the illumination time is *t*, and the initial degradation concentrations after adsorption equilibrium, respectively.

### 2.4. Carrier Migration Measurement

The carrier migration measurements were taken using the standard three-electrode system, with a CS310H electrochemical workstation. First, the 10 mg samples were mixed ultrasonically with 30 μL of 5% Nafion and 5 mL ethanol. Next, 150 μL of ink was coated onto ITO glass with a size of 1 cm × 1 cm as the working electrode. The Pt plate and saturated calomel electrode were used as counter electrode and reference electrode, respectively. The photocurrent performance and Mott–Schottky were measured in 0.1 M Na_2_SO_4_ electrolyte. The photocurrent was measured under 300W Xe light. Electrochemical impedance spectroscopy (EIS) was measured in the 0.1 M KCl solution containing 1 mM Fe(CN)_6_^3−^/Fe(CN))_6_^4−^. The EIS was taken with an amplitude of 10 mV, ranging from 0.01 to 100 MHz.

## 3. Results and discussion

### 3.1. Characterizations of the As-Synthesized Samples

**Figure 1:**
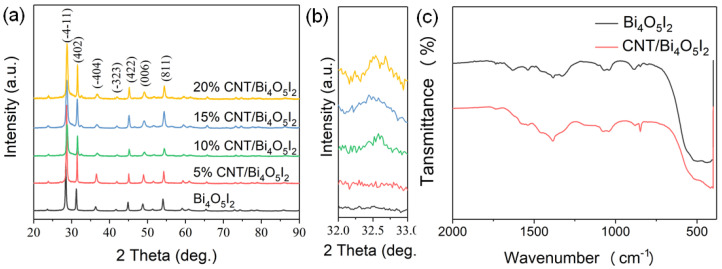
(**a**) XRD patterns, (**b**) the magnification of the region between (402) and (404), and (**c**) FT-IR spectra of the Bi_4_O_5_I_2_ and 15% CNT/Bi_4_O_5_I_2_ composites.

Powder X-ray diffraction (PXRD) was used to investigate the phase composition of Bi_4_O_5_I_2_ and CNT/Bi_4_O_5_I_2_ catalysts. Figure 1a shows that the diffraction pattern of the as-synthesized sample was well indexed to Bi_4_O_5_I_2_ (JCPDS No. 10-0445). No peaks indicating impurities were detected, demonstrating the high purity of the as-obtained catalysts. The diffraction peaks are in reference to the (-4-11), (402), (-404), (-323), (422), (006), (811), (133), (191), and (262) planes, corresponding to the standard diffraction 2θ of the Bi_4_O_5_I_2_ pattern above. After coating with CNT, the diffraction peaks at 32.5° appeared in the composite, suggesting the CNT phase (Figure 1b) [33]. Nevertheless, the typical diffraction peaks of CNT were weak in the CNT/Bi_4_O_5_I_2_ composites (5%, 10%, 15%, 20%), which can be attributed to the low content and high dispersion of CNT in the composites [34]. Fourier transform-infrared spectrometry (FT-IR) was used to analyze the structure of the as-synthesized sample, with or without pristine CNT (Figure 1c). Generally, the stretching vibration of pristine CNT often shows low peak intensity. Hence, the main infrared features of CNT show no obvious or enhanced vibrations. The broad peaks of 500–900 cm^−1^ are ascribed to Bi–O and I–O stretching vibration of Bi_4_O_5_I_2_, decorated onto CNT.

**Figure 2:**
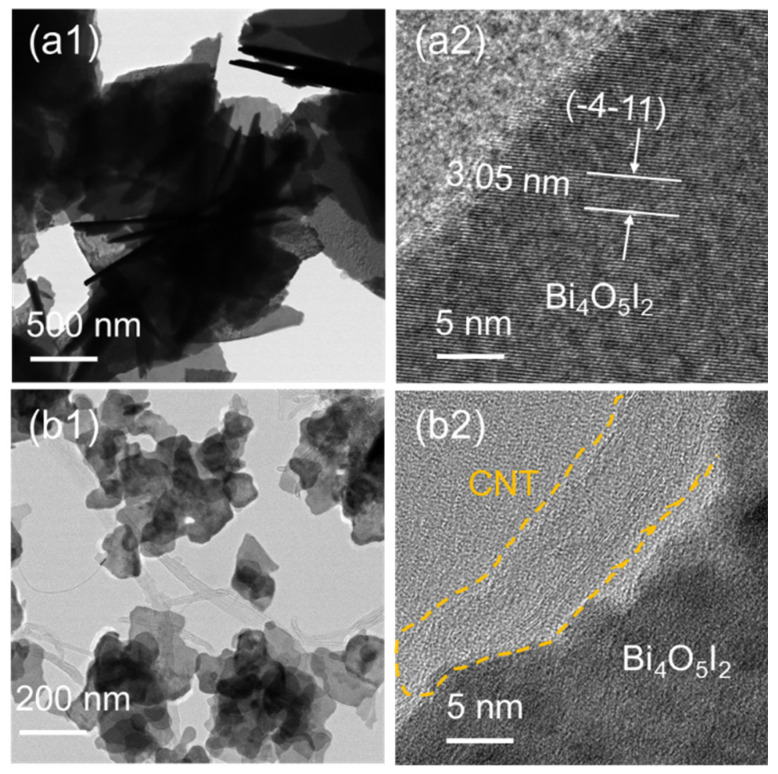
HRTEM images of (**a1**,**a2**) Bi_4_O_5_I_2_ and (**b1**,**b2**) 15% CNT/Bi_4_O_5_I_2_ composite.

Typical TEM images of the Bi_4_O_5_I_2_ and 15% CNT/Bi_4_O_5_I_2_ samples are shown in Figure 2. As shown in Figure 2(a1), the Bi_4_O_5_I_2_ displays flower-like hierarchical nanostructures with a diameter of about 1 μm, constructed with plenty of nanosheets. As shown in Figure 2(a2), the lattice spacing of 0.305 nm matches well with the (-4-11) plane corresponding to Bi_4_O_5_I_2_. Figure 2(b1,b2) show the low- and high-resolution TEM images of 15% CNT/Bi_4_O_5_I_2_. They clearly show that Bi_4_O_5_I_2_ nanosheets are distributed on the framework of CNT, with about a 7-nm width in CNT/Bi_4_O_5_I_2_ (Figure 2(b1)). As shown in Figure 2(b2), the CNT interacts with Bi_4_O_5_I_2_, and the lattice spacing of 0.305 nm is consistent with that of pure Bi_4_O_5_I_2._

**Figure 3:**
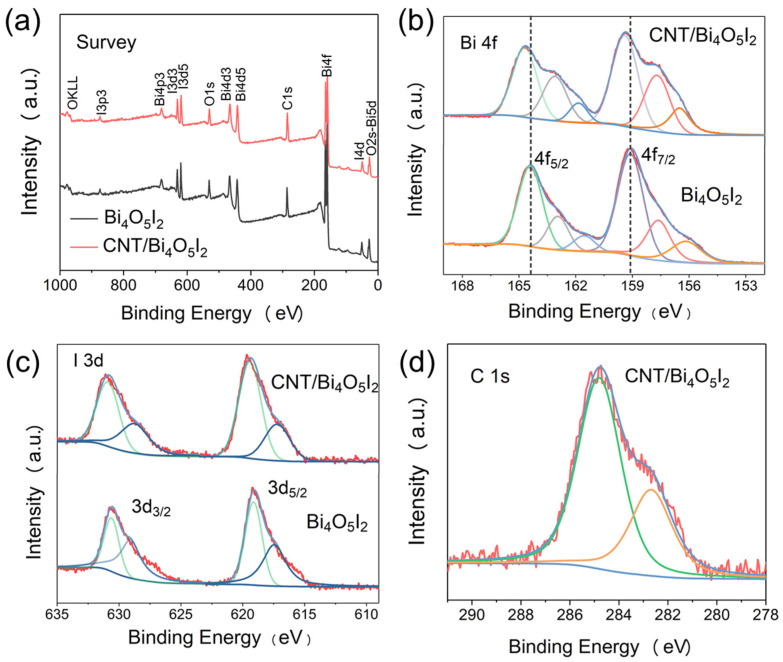
XPS spectra of the as-synthesized Bi_4_O_5_I_2_ nanosheets and CNT/Bi_4_O_5_I_2_: (**a**) survey of the samples, (**b**) Bi 4f, (**c**) I 3d, (**d**) C 1s, respectively.

The chemical states of the as-prepared pure Bi_4_O_5_I_2_ and 15% CNT/Bi_4_O_5_I_2_ were further probed by X-ray photoelectron spectroscopy (XPS) (Figure 3). The low-resolution spectra of pure Bi_4_O_5_I_2_ show obvious Bi, O, I core level and C elements arising from extra carbon-based pollution. In addition, the hybrid catalysis exhibits distinct Bi, O, I, and C core levels, indicating the combination of Bi_4_O_5_I_2_ and CNT. As shown in Figure 3b, the Bi 4f displays Bi 4f_7/2_ (159.1 eV) and Bi 4f_5/2_ (164.4 eV) peaks, which agrees with the previous report [35]. Further deconvolution analysis demonstrates that the Bi^0^ region in pure Bi_4_O_5_I_2_ consists of three peaks at 164.4, 162.9, and 161.5 eV, which are attributed to Bi^3+^, Bi^0^, and a satellite peak, respectively. The presence of Bi^0^ is caused by oxygen vacancy. Moreover, due to the electron-withdrawing ability of CNT, the corresponding Bi peaks are a slightly more positive shift of 0.2 eV in hybrid CNT/Bi_4_O_5_I_2_ than that of pure Bi_4_O_5_I_2_, and the Bi^0^ peak area has become bigger [36]. The I 3d spectra can be deconvoluted into two main peaks centered at 619.1 and 630.5 eV in pure Bi_4_O_5_I_2_ (Figure 3c), which can be attributed to the I 3d_2/3_ and Mo 3d_5/2_, respectively, corresponding to the I^-^ of Bi_4_O_5_I_2_. As displayed in Figure 3d, the C 1s region includes the C-C of CNT and C-O between CNT and Bi_4_O_5_I_2_ [37].

### 3.2. Piezo-and Piezophoto-Catalytic Performances

**Figure 4:**
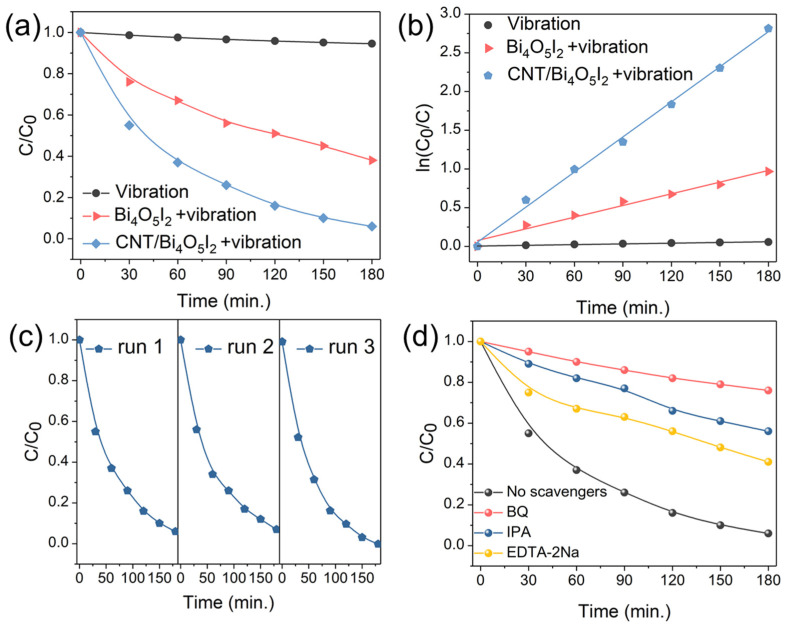
(**a**) Piezocatalytic performances of the pure Bi_4_O_5_I_2_ and 15% CNT/Bi_4_O_5_I_2_ composite on the degradation of RhB under ultrasonic vibration; (**b**) the corresponding *k* values of first-order kinetics plot of Bi_4_O_5_I_2_ and CNT/Bi_4_O_5_I_2_ composite; (**c**) cycling runs of RhB degradation by CNT/Bi_4_O_5_I_2_; (**d**) scavenger trapping experiments of CNT/Bi_4_O_5_I_2_ on the degradation of RhB under ultrasonic vibration.

The piezo-catalytic activities of Bi_4_O_5_I_2_ and CNT/Bi_4_O_5_I_2_ were probed by the representative organic dye RhB under ultrasonic waves. As shown in Figure S1, the 15% CNT/Bi_4_O_5_I_2_ shows the highest piezocatalytic activity among the as-prepared CNT/Bi_4_O_5_I_2_ composites. There is no significant degradation of RhB under ultrasonic vibration without catalysts (Figure 4a). Remarkably, the destruction rate of RhB in pure Bi_4_O_5_I_2_ nanosheets achieves 62% within 3 h, which should be attributed to the piezo-induced positive/negative charges. It is noteworthy that the piezocatalytic performance of Bi_4_O_5_I_2_ nanosheets is still weak, and there is great scope to upgrade this for optimizing the release of strain-induced charges. Therefore, CNT/Bi_4_O_5_I_2_ was designed and evaluated by RhB. As shown in Figure 4a, the removal performance of Bi_4_O_5_I_2_ can be improved coated a typical conductor with CNT, with a 96% degradation rate within 3 h, suggesting that the piezo-generated positive/negative carriers could be promoted to release and play a key role in the degradation efficiency of organic dyes. Moreover, the corresponding kinetics rates reached 0.0003, 0.005, and 0.015 min^−1^. The *k* value of CNT/Bi_4_O_5_I_2_ is 3 times that of Bi_4_O_5_I_2_ under ultrasonic vibration (Figure 4b). In addition, the piezo-stability of CNT/Bi_4_O_5_I_2_ was demonstrated by circulation experiments over 3 serial cycles. As displayed in Figure 4c, the piezo-catalytic activity of the CNT/Bi_4_O_5_I_2_ kept steady, signaling that the as-synthesized hybrid piezo-catalyst is stable under mechanical stress.

To confirm the active species of the piezo-degradation process in 15% CNT/Bi_4_O_5_I_2_, the trapping test was executed [38]. Isopropyl alcohol (IPA), EDTA-2Na, and benzoquinone (BQ) were used as scavengers to trap •OH, holes, and •O_2_^−^, respectively. As demonstrated in Figure 4d, the piezo-degradation performance of RhB in CNT/Bi_4_O_5_I_2_ was slightly restrained with EDTA-2Na scavenger. In contrast, it was significantly repressed with the addition of IPA and BQ. The above results reveal that •OH and •O_2_^−^ are the main active oxidative groups. Under ultrasonic waves, the CNT/Bi_4_O_5_I_2_ piezo-catalyst could produce positive/negative pairs. The negative charges could consume dissolved O_2_ to generate •O_2_^−^ species. Meanwhile, the positive charges could react with H_2_O to supply an •OH group. Then, the active oxidative •OH and •O_2_^−^ species can remove the representative organic dye RhB. For the piezo-degradation of RhB, the holes show the least contribution.

**Figure 5:**
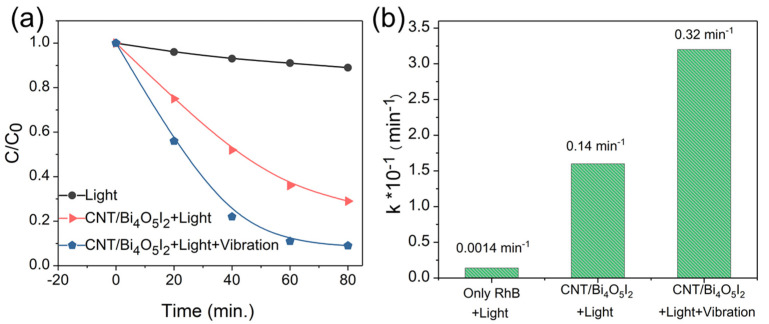
(**a**) Piezo-photocatalytic performances of CNT/Bi_4_O_5_I_2_ composite on the degradation of RhB under different conditions; (**b**) the column chart of the corresponding k values of the CNT/Bi_4_O_5_I_2_ composite.

The piezo-photocatalytic activities of 15% CNT/Bi_4_O_5_I_2_ were also probed by RhB aqueous solution, under an ultrasonic wave or simulated solar light. Firstly, the removal rate of RhB in hybrid CNT/Bi_4_O_5_I_2_ achieved only 11% and 70% within 80 min under simulated solar light and mechanical vibration, respectively, whereas it dramatically reaches 91% with both ultrasonic waves and simulated solar light (Figure 5a). Furthermore, the corresponding kinetics rates reached 0.00014, 0.014, and 0.032 min^−1^. The *k* value under mechanical stress is about 2.3 times that of solar light (Figure 5b), indicating that a strain-induced internal electric field can improve the separation of photoinduced electrons and holes, which is in accordance with the previous reports [23].

### 3.3. Catalytic Mechanism

**Figure 6:**
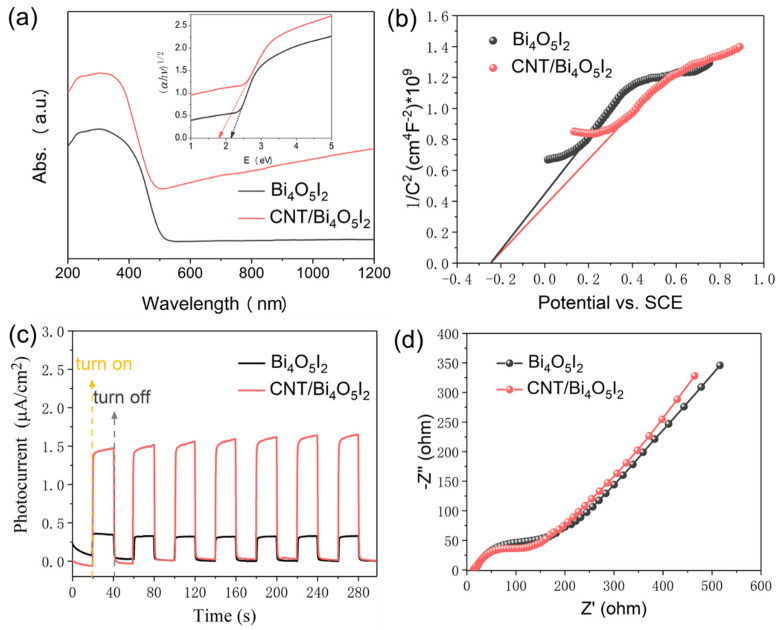
(**a**) UV-vis diffuse reflectance absorption (DRS) spectra of pure Bi_4_O_5_I_2_ and CNT/Bi_4_O_5_I_2_ (inset: estimated band gaps of pure Bi_4_O_5_I_2_ and CNT/Bi_4_O_5_I_2_, respectively); (**b**) Mott–Schottky (M-S) plots, (**c**) transient photocurrent density, (**d**) electrochemical impedance spectroscopy of Bi_4_O_5_I_2_ and CNT/Bi_4_O_5_I_2_.

In general, the band structure of as-prepared catalysts is a key factor for piezo-catalytic activity. Thus, the optical band gaps of pure Bi_4_O_5_I_2_ and CNT/Bi_4_O_5_I_2_ were measured by the UV-vis diffuse reflectance absorption (DRS) spectra. As shown in Figure 6a, the band gaps are 1.84 (Bi_4_O_5_I_2_) and 2.13 eV (CNT/Bi_4_O_5_I_2_), respectively, using Tauc’s equation of *αhv = A*(*hv* − *E*_g_)*^n/2^* [39]. The Mott–Schottky (M-S) measurements of Bi_4_O_5_I_2_ and CNT/Bi_4_O_5_I_2_ are displayed in Figure 6b. The flat-band potential of Bi_4_O_5_I_2_ and CNT/Bi_4_O_5_I_2_ are both −0.24 V (0 V vs. SCE). Thus, the conduction band edge of Bi_4_O_5_I_2_ and CNT/Bi_4_O_5_I_2_ are −0.34 eV (vs. NHE). In addition, the valence band edge of Bi_4_O_5_I_2_ and CNT/Bi_4_O_5_I_2_ are 1.5 eV and 1.79 eV, respectively. Based on the above analysis, the band gaps of these catalysts show a minor effect on the piezocatalytic performance. To further probe the mechanism, the charge transfer processes in Bi_4_O_5_I_2_ and CNT/Bi_4_O_5_I_2_ were explored by transient photocurrent density (PC) and electrochemical impedance spectroscopy (EIS) (Figure 6c,d). Under simulated solar irradiation, the increase in photocurrent in CNT/Bi_4_O_5_I_2_ (1.63 μA·cm^−2^) is much higher than that in Bi_4_O_5_I_2_ (0.32 μA·cm^−2^), indicating efficient charge separation with CNT, used as the carriers sink. Moreover, the CNT/Bi_4_O_5_I_2_ shows a smaller radius of the semicircular Nyquist plot than that of pure Bi_4_O_5_I_2_, which demonstrates more photoinduced charge transfer, due to the addition of CNT.

## 4. Conclusions

In summary, the organic dye (RhB) has been removed by Bi_4_O_5_I_2_ piezocatalysis, and subsequently enhanced by hybrid CNT/Bi_4_O_5_I_2_ under ultrasonic vibration or simulated solar light. In addition, the cycling test revealed that CNT/Bi_4_O_5_I_2_ maintains good stability. Importantly, we found that the strain-induced internal electric field via the piezoelectric effect can boost the separation of photoinduced electron/hole pairs. In addition, the piezo-induced positive/negative charge of Bi_4_O_5_I_2_ could be released more easily, making good use of the excellent electroconductibility of CNT. Our results may offer promising approaches to sketching efficient piezo-photocatalysis for the full utilization of solar energy or mechanical vibration.

## Data Availability

Not applicable.

## Supplementary Materials

The following are available online at https://www.mdpi.com/article/10.3390/ma14164449/s1, Figure S1: Piezocatalytic performances of CNT/Bi_4_O_5_I_2_ composites (5%, 10%, 15%, 20%) on the degradation of RhB.
