# Supplementary material for: Boosting Piezo/Photo-Induced Charge Transfer of CNT/Bi4O5I2 Catalyst for Efficient Ultrasound-Assisted Degradation of Rhodamine B
Source: Materials (Basel). 2021 Aug 9;14(16):4449. doi: 10.3390/ma14164449 (PMC8401989; doi:10.3390/ma14164449)
Supplement: Supplementary file 1 [file materials-14-04449-s001.zip › materials-1233763-supplementary.pdf]

Supplementary Data

for

Boosting Piezo/photo-induced Charge Transfer of $CNT/Bi_4O_5I_2$ Catalyst for Efficient Ultrasound-assisted Degradation of Rhodamine B

Yang Wang [1,†], Dongfang Yu [2,*,†], Yue Liu [2], Xin Liu [2] and Yue Shi [1]

[1] Chang Wang School of Honors, Nanjing University of Information Science and Technology, Nanjing 210044, China; wangyang-cw@nuist.edu.cn (Y.W.); shiyue@nuist.edu.cn (Y.S.)

[2] School of Environmental Science and Engineering, Nanjing University of Information Science and Technology, Nanjing 210044, China; liuy@nuist.edu.cn (Y.L.); lx@nuist.edu.cn (X.L.)

* Correspondence: yudongfang@nuist.edu.cn

† These authors contributed equally to this work.

Figures

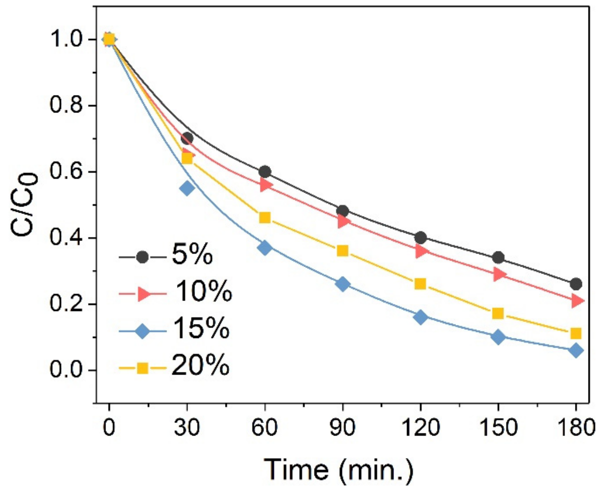

Figure S1 Piezocatalytic performances of $CNT/Bi_4O_5I_2$ composites (5%, 10%, 15%, 20%) on the degradation of RhB.
